# Lives of Eminent American Physicians and Surgeons of the Nineteenth Century

**Published:** 1861-01

**Authors:** 


ART. XII.—*Lives of Eminent American Physicians and Surgeons of the Nineteenth Century.* Edited by S. D. GROSS, M.D., etc., etc. 8vo., pp. 836. Lindsay & Blakiston: Philadelphia, 1861.

THIS long-promised work has at length been issued from the press, in a style highly creditable to all concerned. It comprises thirty-two memoirs of distinguished physicians and surgeons, and will, we feel satisfied, be read with the deepest interest, not only by medical men, but by lawyers, divines, and the more intelligent portion of the public generally. Such a work has long been needed, and it cannot fail to be hailed by the profession throughout the country as a valuable addition to its literature. As a more extended notice of it will appear in a future number of the REVIEW, we content ourselves at present with announcing the fact of its publication, and the expression of our conviction that it should be in the library of every intelligent physician.

